# Seeing invisible objects with intelligent optics

**DOI:** 10.1038/s41377-024-01575-2

**Published:** 2024-09-05

**Authors:** Isaac Nape, Andrew Forbes

**Affiliations:** School of Physics, University of the Witwatersrand, Johannesburg, South Africa

**Keywords:** Optical physics, Optical techniques

## Abstract

Transparent objects are invisible to traditional cameras because they can only detect intensity fluctuations, necessitating the need for interferometry followed by computationally intensive digital image processing. Now it is shown that the necessary transformations can be performed optically by combining machine learning and diffractive optics, for a direct in-situ measurement of transparent objects with conventional cameras.

Recent advances in optical imaging systems have greatly enhanced our ability to capture images at the microscopic level with exceptional resolution and speed, driving progress in fields such as biological research and metrology. While these advancements have simplified the imaging of complex surface profiles, imaging transparent objects remains a challenge. This is because camera sensors are only capable of detecting fluctuations in light intensity. As a result, cells and microbes often go undetected as they allow light to pass through without significant scattering. The primary issue is that transparent objects encode information in the phase of light—a property that is invisible to standard cameras and remains a largely elusive property to detect directly.

Addressing this challenge, Jingxi Li and colleagues have recently introduced a diffractive imager^1^ that is capable of converting phase information into amplitude information, detectable as intensity fluctuations (see the inset in Fig. 1). This innovative approach allows for the direct imaging of the refractive index properties (the extent to which light slows down in a material) of objects, offering a method that is far simpler and more effective than previous techniques. This technique now makes the phase of an optical field visible to standard cameras without the need for interferometry and digital image processing.

**Fig. 1:**
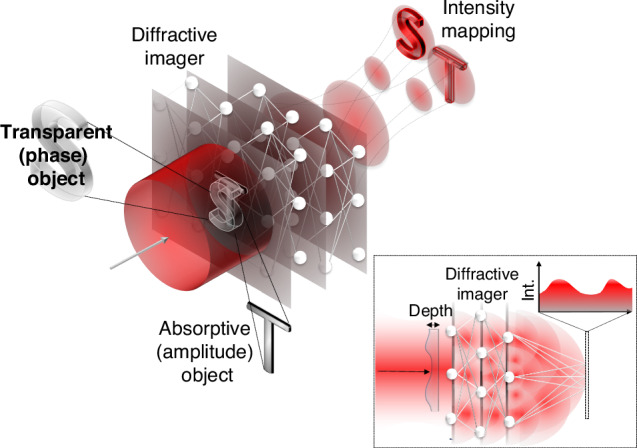
An all-optical neural network for phase imaging. The behaviour of optical waves are dominated by phase, for instance, the evolution of optical fields and images during propagation is based on interference of the many components that make up the wave, where phase variation is converted into intensity variation. This principle can be enhanced with a series of diffractive optical elements to maximise this mapping. When trained with machine learning, the result is a phase and amplitude imager using a conventional camera without the need for reference beams or digital signal processing

In conventional approaches, a phase measurement relies on indirect methods^2^ involving interferometers, projective filters, and iterative techniques, which were seen as the most reliable means for obtaining quantitative phase measurements^3^. In the interferometric methods, a known reference field is superimposed on a field carrying unknown phase information, exploiting the constructive and destructive interference of optical waves to convert phase variations into visible patterns of light and dark fringes. These fringes contain critical data about transparent objects such as their refractive index, but unravelling the concealed phase profile hidden beneath the amplitude and intensity variations of the measured interferogram is a complex task, often addressed using iterative phase retrieval algorithms^4^ which can be computationally costly and may introduce numerical artifacts hampering accuracy.

The innovative demonstration by Jingxi Li et al. overcomes the need for secondary interference measurements with reference fields and the application of post-reconstruction algorithms. Instead, it exploits the self-interference to realize the conversion process. Insight from centuries earlier, following the work of Ernst Abbe, can help understand why this works. Abbe theorized that optical images are essentially interference patterns because parts of a field interfere with themselves in order to produce the observed complicated patterns – this is what we typically see in free-space diffraction or in Thomas Young’s double slit experiment where a field self-interferes even at the single photon level. With modern optics, this self-interference can be realized with unprecedented control.

In the reported work, the authors exploit self-interference, harnessed using specially constructed multiple diffraction screens to achieve the desired mappings that mediate the convention from phase to amplitude. The conversion process can be thought of as a calculation that light performs as it propagates such as to conduct a mapping from input phases onto output intensities. This computation takes place as the light field is propagated through a series of *intelligently* designed diffractive optics. To construct these diffractive masks, the team utilized a supervised learning approach to optimize the diffractive layers to carry out the complex transformation required for accurate mapping. Remarkably, these layered diffractive optical elements function similarly to layers in an artificial neural network, trained to perform intricate operations at the speed of light. Thus, their method represents a unique optical implementation of a complicated network that resembles an artificial neural network, harnessing artificial intelligence entirely within an optical system, a topic that has seen many advances of late^5–7^.

This remarkable feet indeed hinges on the recent progress that has been made in Artificial intelligence (AI). It has become a crucial element in the workflow of modern optics labs, assisting in the design of novel optical elements^8^, and modern measurement schemes^9^ and assisting in the discovery of new experiments^10^. In phase imaging, it has been used as a tool to accelerate the phase retrieval process. In such settings, a neural network would be trained on a graphics processing unit (GPU) using sets of example inputs—interferograms—and their corresponding profiles. In the end the network learns the function that solves the problem. This method, although demanding in terms of computational resources and data requirements, allows for the efficient learning of complex nonlinear transformations also unveils phase profiles from complicated images of interference patterns.

The present work moves the topic of all-optical artificial intelligence in a new direction. Their diffractive network interacts directly with the optical field enabling phase information to flow through through the diffractive network via free space diffraction of light. This can also be explained by Huygens principle, where each element within this network acts as a node, propagating secondary wavelets forward to the subsequent diffractive optic in the sequence. This physical realization of such diffractive neural networks illustrates a profound shift towards integrating artificial intelligence directly within the operational framework of optical systems.

In their demonstration, the authors do not only measure phase, but are also able to measure the amplitude information via a multiplexing approach. This can be done in three ways: (i) multiplexing through space and path encoding, as shown in Fig. 1, where the phase and amplitudes are separated in space. (ii) using wavelength multiplexing where the amplitude and phase share the same path but are encoded on separate wavelengths; (iii) combining both approaches, so that the amplitudes and phases are separated in space but at different wavelengths. These diverse approaches are made possible by the ability to encode information into numerous degrees of freedom of light – structured light – for the enhanced functionality of light in numerous applications^11^.
